# Association of Urinary Metal Profiles with Altered Glucose Levels and Diabetes Risk: A Population-Based Study in China

**DOI:** 10.1371/journal.pone.0123742

**Published:** 2015-04-13

**Authors:** Wei Feng, Xiuqing Cui, Bing Liu, Chuanyao Liu, Yang Xiao, Wei Lu, Huan Guo, Meian He, Xiaomin Zhang, Jing Yuan, Weihong Chen, Tangchun Wu

**Affiliations:** Key Laboratory of Environment and Health, Ministry of Education & Ministry of Environmental Protection, and State Key Laboratory of Environmental Health (Incubation), School of Public Health, Tongji Medical College, Huazhong University of Science and Technology, 13 Hangkong Rd, Wuhan 430030, China; University of Catanzaro Magna Graecia, ITALY

## Abstract

**Background:**

Elevated heavy metals and fasting plasma glucose (FPG) levels were both associated with increased risk of cardiovascular diseases. However, studies on the associations of heavy metals and essential elements with altered FPG and diabetes risk were limited or conflicting. The objective of this study was to evaluate the potential associations of heavy metals and essential trace elements with FPG and diabetes risk among general Chinese population.

**Methods:**

We conducted a cross-sectional study to investigate the associations of urinary concentrations of 23 metals with FPG, impaired fasting glucose (IFG) and diabetes among 2242 community-based Chinese adults in Wuhan. We used the false discovery rate (FDR) method to correct for multiple hypothesis tests.

**Results:**

After adjusting for potential confounders, urinary aluminum, titanium, cobalt, nickel, copper, zinc, selenium, rubidium, strontium, molybdenum, cadmium, antimony, barium, tungsten and lead were associated with altered FPG, IFG or diabetes risk (all P< 0.05); arsenic was only dose-dependently related to diabetes (P< 0.05). After additional adjustment for multiple testing, titanium, copper, zinc, selenium, rubidium, tungsten and lead were still significantly associated with one or more outcomes (all FDR-adjusted P< 0.05).

**Conclusions:**

Our results suggest that multiple metals in urine are associated with FPG, IFG or diabetes risk. Because the cross-sectional design precludes inferences about causality, further prospective studies are warranted to validate our findings.

## Introduction

Elevated fasting plasma glucose (FPG) is an independent risk factor for type 2 diabetes and has been linked to increased risk of cardiovascular disease (CVD) and mortality [1,2]. Epidemiological evidence has suggested that toxic heavy metals, including arsenic, cadmium, antimony and lead, are associated with an increased risk of CVD [3,4]. However, evidence for the association of the metals with FPG and diabetes is limited or conflicting. Studies conducted in Taiwan and Bangladesh showed that exposure to high arsenic levels via drinking water was consistently associated with increased risk of diabetes [5]; while other studies did not find a significant association for lower level arsenic [6,7]. In addition, Navas-Acien et al. [8,9] suggested that elevated urinary arsenic level was significantly related to increased risk of diabetes whereas results from another study [10] found the association to be null. Moreover, Schwartz et al. [11] reported that urinary cadmium was associated with impaired glucose regulation and diabetes among general U.S. population, while Swaddiwudhipong et al. [12] did not find association between urinary cadmium and diabetes in a Thai population. Although exposure to lead in the environment is associated with CVD risk [3], few studies have investigated the association between lead and FPG or diabetes. For example, a recent study conducted in a Korean population indicated that blood lead was not related to the prevalence of diabetes [13]. Antimony was known to be a genotoxic element in vitro and in vivo [14] and a previous study suggested that urinary antimony was associated with CVD in NHANES1999-2006 participants [4]. Nonetheless, as far as we know, no prior study has investigated the relationship between antimony and diabetes outcomes.

Trace elements such as vanadium, chromium, manganese, iron, cobalt, nickel, copper, zinc, selenium and molybdenum are involved in various metabolic characteristics and biological functions [15–17]. These element deficiencies or excesses are frequently related to human diseases. Alterations in the status of the trace elements could stem from chronic uncontrolled hyperglycemia, and on the other hand, some of these nutrients can directly modulate glucose homeostasis [18,19]. However, few epidemiological studies have been conducted to examine the association of FPG and diabetes with the trace elements except zinc and selenium. Accumulating evidence has suggested that zinc supplementation has beneficial effects on glycaemic control in diabetic patients [20]; however, few studies have investigated the relationship between zinc and FPG among general population. Moreover, whether elevated selenium intake was associated with reduced risk of diabetes or not still remained to be investigated [21,22].

The exact physiological roles of the aluminum, titanium, rubidium, strontium, tin, barium, tungsten, thallium and uranium in the human body are unclear. Furthermore, there was lacking epidemiological evidence of associations between environmental exposure to these chemicals and the health outcomes. However, studies in vivo and vitro have shown that these metals may participant in pathophysiology processes through oxidative stress [23–31], which was considered to be involved in the development of diabetes and CVD [32].

Based on this background, in the present study we aimed to examine the associations of FPG and impaired fasting glucose (IFG) and diabetes risk with the urinary levels of 23 nutrient elements and toxic heavy metals as well as other metals including aluminum, titanium, vanadium, chromium, manganese, iron, cobalt, nickel, copper, zinc, arsenic, selenium, rubidium, strontium, molybdenum, cadmium, tin, antimony, barium, tungsten, thallium, lead and uranium among 2242 Chinese adults in a resident community.

## Materials and Methods

### Study Population

As described in our previous study [33], the source population of this study consisted of 3053 community residents aged 18–80 years, who resided in Wuhan city for at least 5 years. Recruitment took place between April and May 2011 and sampling was performed at local community health centers. All subjects gave written informed consent and the study protocol was approved by the Ethics and Human Subject Committee of Tongji Medical College. All subjects were examined after an overnight fast. Information about socio-demographic factors, personal characteristics and medical history was obtained by trained reviewers using a standardized structured questionnaire. In addition, information on a subject’s history of diabetes included questions about prior diagnoses of diabetes by a physician and current use of insulin and oral hypoglycemic drugs. A health examination including measurement of height, weight, and blood pressure was also performed by qualified physicians. Blood samples were drawn from each participant and divided into two sets. One was forwarded to our laboratory within 2 hours of collection for analysis of FPG and other biochemical measurements such as cholesterol and triglycerides. The other sample was stored at -80°C for further analysis of other markers. Morning spot urine samples were also collected from each subject and stored at -20°C until laboratory analysis.

### Exclusion criterion

For the current analyses, we excluded 465 subjects with missing urinary metals and 10 subjects with missing FPG as well as 52 subjects with self-reported nephritis, which may result in abnormal urinary outputs of trace elements [34]. We also excluded 308 participants with abnormal urinary creatinine levels according to the WHO exclusionary guidelines [35]. In addition, 107 subjects were excluded because of missing covariate data (36 missing height or weight, 40 missing systolic or diastolic pressure, 10 missing total cholesterol or triglyceride and 47 missing urinary creatinine). The final study population consisted of 2242 participants.

### Definition of the outcome and confounders

The definitions of normal glucose tolerance (NGT), impaired fasting glucose (IFG) and diabetes met the respective diagnostic criteria recommended by the American Diabetes Association [36]. NGT was defined as individuals without self-reported diabetes and glycemic control drug use, and with a FPG < 100 mg/dL. IFG was defined as FPG between 100–125 mg/dL, absence of previously diagnosed diabetes, and absence of glycemic control medications. Diabetes was diagnosed with a fasting glucose concentration >125 mg/dL or a self-reported physician diagnosis of diabetes, or self-reported use of insulin or oral hypoglycemic medication.

One pack year is defined as 20 cigarettes smoked every day for one year. Body mass index (BMI) was calculated as weight in kilograms divided by height in meters squared. Hypertension was defined as either having a systolic blood pressure that is greater than or equal to 140 mmHg or a diastolic blood pressure that is greater than or equal to 90 mmHg, or having been diagnosed with hypertension by a physician. The definition of hyperlipidemia was total cholesterol of more than 220 mg/dL, triglycerides of more than 150 mg/dL, or having been diagnosed with hyperlipidemia by a physician.

### Determination of FPG

FPG was analyzed by the enzymatic colorimetric method on a fully-automated biochemical analyzer RX daytona (Randox Laboratories Ltd., UK). The experiment was carried out according to the standard operation procedure provided by the manufacture. Furthermore, internal quality control samples (Randox kits) with every batch of samples were analyzed after standardization.

### Measurement of urinary metal concentrations

The determination of metal contents in urine was performed as previously described [37], with minor modification. In brief, the frozen urine samples were completely thawed at room temperature and homogenized. A 3.0 ml aliquot of urine was transferred to a polypropylene tube (Jiayu experiment instrument Co., Ltd., Haimen, China) containing 15.0 μl of 67% (v/v) HNO_3_ and stored in a refrigerator at 5°C. Two hours before sample preparation the urine samples were brought to room temperature. A 1.0 ml of the sample was pipetted into a 10ml disposable polypropylene tube and then filled up to 5.0 ml with 1.2% (v/v) HNO_3_ (OptimaTM grade, Fisher, Belgium) using adjustable volume pipette samplers. The samples were then measured using an inductively coupled plasma mass spectrometry with an octopole based collision/reaction cell (Agilent 7700 Series, Waldbronn, USA).

### Quality control procedures

For quality control of urinary metal measurements, duplicate analysis, spiked pooled sample (randomly collected from 100 samples) and NIST SRM 2670a (toxic elements in urine) as well as NIST SRM 1640a (consisting of trace elements in natural water) were used [33]. The relative standard deviation (RSD) of the duplicate analyses (three times) for the 23 metals in each urine sample was calculated to assess the accuracy. The concentration of the metal was re-quantified if the RSD was greater than 10%. In addition, we used spiked recoveries of the pooled urine to evaluate the accuracy of method for determination of titanium, iron, rubidium, and strontium since no certified standards exist for these elements. The spiked recoveries for these metals were in the range 78.3–113.2%. We also used SRM 2670a to verify method accuracy of manganese, cobalt, selenium, molybdenum, cadmium, antimony, thallium, lead and uranium. We estimated the determination accuracy of these metals by comparing the difference between the certified values available and the measured values with their uncertainty using the previous method reported by Linsinger [38]. The measurement results by our method were in agreement with the SRM 2670a certified values. Aluminum, vanadium, chromium, nickel, copper, zinc, arsenic, tin, barium and tungsten in SRM 2670a were not certified; nonetheless, the NIST provided reference or information values. The mean results of aluminum, vanadium, chromium, nickel, copper, zinc, arsenic, tin and barium by the method agreed within 10.7% of the target value. For tungsten, we determined a concentration of 0.6 μg/L, and the information value provided by NIST was < 1.0 μg/L. At the same time, SRM 1640a was always analyzed after every 20 samples to ensure instrument performance, which was certified for all metals except for titanium, rubidium, tin, antimony and tungsten. The check standards were used to compare metals if their concentrations were not in agreement with actual concentrations of SRM 1640a. The instrument was recalibrated using multi-element standards and the previous 20 samples were reanalyzed if their concentrations were significantly different from actual concentrations.

The limits of quantification (LOQ) for the urinary metals were in the range 0.0004–0.292 μg/L. We replaced the metal concentrations below the LOQ with LOQ/2. We reported the mean of three replicate measurements for each metal concentration in urine.

### Determination of creatinine

Urinary creatinine was determined by the picric acid assay on a fully automated clinical chemistry analyzer (Mindray Medical International Ltd., Shenzhen, China).

### Statistical Analysis

Descriptive statistics were calculated for all demographic and clinical characteristics of the study subjects. FPG data was logarithmically transformed to reduce their skewness. We used generalized linear models (GLMs) to estimate associations between FPG and urinary metal concentrations by quartiles. The odds ratio (OR) and 95% confidence intervals (CIs) for the risks of diabetes and IFG in relation to urinary metals were also estimated using logistic regression models (LRMs). The change-in-effect estimate method was used to identify confounding variables [39]. Potential confounders were included into the regression model if they changed the effect estimates by greater than or equal to 10% for at least one metal exposure [40]. Finally, we included age, pack year, BMI as continuous variables; gender, hypertension, hyperlipidemia, family history of diabetes, anti-diabetes drug and insulin use as dichotomous variables; and smoking status and alcohol intake as dummy variables in each model. Creatinine corrected spot urinary concentrations for adjusting dilution have been suggested as good surrogate measures of 24-hour urine excretion of chemical substances. However, the analysis of association using regression models included the creatinine-adjusted chemical concentrations as an independent variable may introduce potential bias [41,42]. Therefore, in the present study, we included the creatinine concentration as a continuous covariate in the models according to the recommendation [41].

All analyses were conducted using SPSS version 12.0. A two-tailed P-value below 5% was considered significant. We also used the positive false discovery rate (FDR) method to adjust for multiple comparisons. The FDR-adjusted P-value was calculated from 23 hypothesis tests using the spreadsheet software based on a previous study [43].

## Results

### Basic characteristics of study participants

Participant characteristics are shown in Table 1. The prevalence of diabetes and IFG was 9.7% and 11.6% among the 2242 subjects respectively. The mean age was 51.5, 57.1, 60.1 and 53.0 years for NGT and IFG subjects, diabetics and total population, respectively. On average, there were more female participants than male participants among each sub-group, as well as in total population. Most of our population (75.1%) had never smoked.

**Table 1:** Basic characteristics and clinical parameters of subjects in communities of Wuhan city, China.

Variables	Subjects with NGT (n = 1765)	Subjects with IFG (n = 259)	Subjects with diabetes (n = 218)	Total (n = 2242)
Age, year	51.5 ± 13.4	57.1 ± 10.4	60.1 ± 10.1	53.0 ± 13.2
Gender				
Male	580 (32.9)	110 (42.5)	89 (40.8)	779 (34.7)
Female	1185 (67.1)	149 (57.5)	129 (59.2)	1463 (65.3)
BMI, kg/m^2^	23.8 ± 3.4	25.4 ± 3.4	25.4 ± 3.4	24.2 ± 3.4
Smoking status				
Never	1352 (76.6)	180 (69.5)	152 (69.7)	1684 (75.1)
Former	74 (4.2)	24 (9.3)	30 (13.8)	128 (5.7)
Current	339 (19.2)	55 (21.2)	36 (16.5)	430 (19.2)
Pack year	26.2 ± 22.4	28.3 ± 21.3	31.4 ± 28.3	27.1 ± 23.0
Alcohol use				
Never	1404 (79.5)	178 (68.7)	177 (81.2)	1759 (78.5)
Former	55 (3.1)	15 (5.8)	16 (7.3)	86 (3.8)
Current	306 (17.3)	66 (25.5)	25 (11.5)	397 (17.7)
FPG, mg/dL	84.1 ± 8.8	107.9 ± 6.6	141.5 ± 57.0	92.4 ± 26.4
Family history of diabetes				
No	1646 (93.3)	240 (92.7)	182 (83.5)	2068 (92.2)
Yes	119 (6.7)	19 (7.3)	36 (16.5)	174 (7.8)
Oral anti-diabetes drugs				
No	1765 (100.0)	259 (100.0)	112 (51.4)	2136 (95.3)
Yes	-	-	106 (48.6)	106 (4.7)
Insulin use				
No	1765 (100.0)	259 (100.0)	203 (93.1)	2227 (99.3)
Yes	-	-	15 (6.9)	15 (0.7)
Hypertension				
No	1140 (64.6)	110 (42.5)	79 (36.2)	1329 (59.3)
Yes	625 (35.4)	149 (57.5)	139 (63.8)	913 (40.7)
Hyperlipidemia				
No	1096 (62.1)	104 (40.2)	65 (29.8)	1265 (56.4)
Yes	669 (37.9)	155 (59.8)	153 (70.2)	977 (43.6)
Urinary creatinine, mmol/L	12.5 ± 5.7	12.9 ± 5.6	12.6 ± 5.9	12.6 ± 5.7

Abbreviations: BMI, body mass index; FPG, fasting plasma glucose; NGT, normal glucose tolerance; IFG, impaired fasting glucose. Data were presented as mean ± SD or n (%).

### Distribution of urinary metals

The distribution of 23 urinary metals (unadjusted and adjusted for urinary creatinine) is given in S1 Table. The proportion of most metals below the LOQ was less than 1.0%, whereas the ratios of urinary tin, tungsten and lead below the LOQ were 38.0%, 2.9% and 5.6% respectively.

### Urinary metals and FPG

Results of GLMs predicting altered FPG for 23 urinary metals are shown in Table 2. After adjustment for age, gender, BMI, smoking status, pack year, alcohol status, hypertension, hyperlipidemia, family history of diabetes, anti-diabetes drugs, insulin use and urinary creatinine, there were no significant associations of FPG with urinary vanadium, chromium, manganese, cobalt, arsenic, strontium, molybdenum, cadmium, tin, antimony, barium, thallium and uranium. However, we did observe significant dose-response relationships between FPG and urinary output of titanium, zinc, selenium, rubidium, tungsten and lead (all *P*< 0.05). Participants in the fourth quartiles of urinary titanium and zinc, in the third and fourth quartiles of urinary selenium and tungsten, and in the second and fourth quartiles of urinary lead had significantly higher FPG levels compared with subjects in the first titanium, zinc, selenium, tungsten and lead quartiles respectively [β (95% CIs) = 0.039 (0.015, 0.063) for titanium, 0.068 (0.042, 0.093) for zinc, 0.024 (0.000, 0.048) and 0.045 (0.019, 0.072) for selenium, 0.029 (0.007, 0.052) and 0.038 (0.015, 0.062) for tungsten, and 0.026 (0.004, 0.048) and 0.031 (0.008, 0.055) for lead]. Participants in the third and fourth quartiles of urinary rubidium, however, had significant decreases of 0.034 mg/dL (95% CIs: -0.058, -0.010) and 0.039 mg/dL (95% CIs: -0.067, -0.012) in FPG compared with those in the first quartile respectively. Moreover, we found that participants in the third quartile of aluminum, the fourth quartile of nickel and copper had significant increases of 0.024 mg/dL (95% CIs: 0.002, 0.046), 0.023 mg/dL (95% CIs: 0.000, 0.047) and 0.025 mg/dL (95% CIs: 0.001, 0.050) in FPG whereas participants in the fourth quartile of iron had a significant decrease of 0.024 mg/dL (95% CIs: -0.046, -0.001) in FPG compared with those in the first quartile respectively, but there was a lack of dose-response trends. Furthermore, the dose-response associations of FPG with titanium, zinc, selenium, rubidium and tungsten remained significant even after multiple corrections (all FDR-adjusted *P*< 0.05).

**Table 2:** Adjusted regression coefficients (95% CIs) for the association between quartiles of urinary metals and FPG.

Variables	Q1 (Lowest)	Q2	Q3	Q4 (Highest)	*P*	*P* *
**Aluminium**	< 21.37	21.37–31.61	31.62–49.47	> 49.47		
β (95%CIs)	0.000	0.015 (-0.007, 0.037)	0.024 (0.002, 0.046)	0.010 (-0.013, 0.032)	0.317	0.561
**Titanium**	< 26.08	26.08–44.85	44.86–72.02	> 72.02		
β (95%CIs)	0.000	0.018 (-0.004, 0.041)	0.009 (-0.013, 0.032)	0.039 (0.015, 0.063)	0.006	0.028
**Vanadium**	< 0.34	0.34–0.49	0.50–0.74	> 0.74		
β (95%CIs)	0.000	-0.004 (-0.026, 0.018)	-0.008 (-0.031, 0.014)	-0.006 (-0.028, 0.017)	0.580	0.702
**Chromium**	< 0.94	0.94–1.42	1.43–2.22	> 2.22		
β (95%CIs)	0.000	-0.004 (-0.026, 0.018)	-0.004 (-0.026, 0.018)	0.014 (-0.009, 0.036)	0.243	0.466
**Manganese**	< 1.57	1.57–2.44	2.45–3.75	> 3.75		
β (95%CIs)	0.000	0.018 (-0.004, 0.040)	0.020 (-0.002, 0.042)	-0.012 (-0.034, 0.011)	0.357	0.587
**Iron**	< 44.24	44.24–75.57	75.58–139.53	> 139.53		
β (95%CIs)	0.000	-0.013 (-0.035, 0.009)	-0.012 (-0.034, 0.010)	-0.024 (-0.046, -0.001)	0.055	0.158
**Cobalt**	< 0.16	0.16–0.24	0.25–0.40	> 0.40		
β (95%CIs)	0.000	0.003 (-0.020, 0.026)	0.008 (-0.016, 0.032)	0.017 (-0.007, 0.042)	0.156	0.359
**Nickel**	< 1.48	1.48–2.26	2.27–3.52	> 3.52		
β (95%CIs)	0.000	0.006 (-0.016, 0.028)	0.002 (-0.020, 0.025)	0.023 (0.000, 0.047)	0.083	0.212
**Copper**	< 5.19	5.19–7.40	7.41–10.71	> 10.71		
β (95%CIs)	0.000	0.000 (-0.022, 0.023)	0.001 (-0.023, 0.024)	0.025 (0.001, 0.050)	0.053	0.158
**Zinc**	< 168.05	168.05–270.49	270.50–412.36	> 412.36		
β (95%CIs)	0.000	-0.003 (-0.025, 0.019)	0.009 (-0.014, 0.032)	0.068 (0.042, 0.093)	< 0.001	< 0.001
**Arsenic**	< 17.17	17.17–28.43	28.44–46.45	> 46.45		
β (95%CIs)	0.000	-0.020 (-0.043, 0.002)	-0.022 (-0.046, 0.002)	-0.008 (-0.035, 0.019)	0.567	0.702
**Selenium**	< 4.55	4.55–7.49	7.50–11.76	> 11.76		
β (95%CIs)	0.000	0.008 (-0.014, 0.031)	0.024 (0.000, 0.048)	0.045 (0.019, 0.072)	< 0.001	0.003
**Rubidium**	< 1187.58	1187.58–1956.85	1956.86–3035.43	> 3035.43		
β (95%CIs)	0.000	-0.015 (-0.038, 0.008)	-0.034 (-0.058, -0.010)	-0.039 (-0.067, -0.012)	0.002	0.012
**Strontium**	< 75.54	75.54–122.63	122.64–178.05	> 178.05		
β (95%CIs)	0.000	-0.002 (-0.025, 0.020)	0.014 (-0.008, 0.037)	0.012 (-0.012, 0.035)	0.174	0.364
**Molybdenum**	< 27.80	27.80–45.96	45.97–77.95	> 77.95		
β (95%CIs)	0.000	-0.011 (-0.034, 0.011)	-0.017 (-0.040, 0.006)	-0.009 (-0.034, 0.017)	0.440	0.633
**Cadmium**	< 0.53	0.53–0.89	0.90–1.42	> 1.42		
β (95%CIs)	0.000	0.003 (-0.020, 0.025)	-0.003 (-0.027, 0.021)	0.008 (-0.019, 0.035)	0.666	0.756
**Tin**	< LOQ	LOQ—0.30	0.31–0.44	> 0.44		
β (95%CIs)	0.000	0.006 (-0.015, 0.028)	0.013 (-0.010, 0.035)	0.007 (-0.015, 0.030)	0.390	0.598
**Antimony**	< 0.11	0.11–0.16	0.17–0.23	> 0.23		
β (95%CIs)	0.000	-0.002 (-0.025, 0.020)	-0.015 (-0.038, 0.008)	0.010 (-0.016, 0.035)	0.723	0.756
**Barium**	< 2.52	2.52–3.78	3.79–5.77	> 5.77		
β (95%CIs)	0.000	-0.004 (-0.026, 0.018)	-0.008 (-0.031, 0.014)	0.009 (-0.013, 0.032)	0.514	0.695
**Tungsten**	< 0.07	0.07–0.12	0.13–0.21	> 0.21		
β (95%CIs)	0.000	0.005 (-0.017, 0.027)	0.029 (0.007, 0.052)	0.038 (0.015, 0.062)	< 0.001	0.003
**Thallium**	< 0.32	0.32–0.55	0.56–0.86	> 0.86		
β (95%CIs)	0.000	-0.001 (-0.024, 0.022)	-0.001 (-0.025, 0.023)	-0.005 (-0.031, 0.021)	0.718	0.756
**Lead**	< 2.13	2.13–3.18	3.19–4.53	> 4.53		
β (95%CIs)	0.000	0.026 (0.004, 0.048)	0.013 (-0.010, 0.036)	0.031 (0.008, 0.055)	0.035	0.134
**Uranium**	< 0.020	0.020–0.030	0.031–0.047	> 0.047		
β (95%CIs)	0.000	0.006 (-0.016, 0.029)	-0.007 (-0.030, 0.016)	0.003 (-0.020, 0.026)	0.904	0.904

Abbreviations: FPG, fasting plasma glucose. All models were adjusted for age, gender, BMI, smoking status, pack year, alcohol status, hypertension, hyperlipidemia, family history of diabetes, anti-diabetes drugs, insulin use and urinary creatinine.

*FDR-adjusted.

### Urinary metals and diabetes

Urinary aluminum, titanium, vanadium, chromium, manganese, iron, cobalt, rubidium, strontium, cadmium, tin, barium, thallium, lead and uranium were not associated with diabetes risk (Table 3). However, we observed statistically significant correlations of the fourth quartiles of nickel, copper, arsenic, molybdenum and antimony as well as the third and fourth quartiles of zinc and tungsten with increased diabetes risk (Table 3). After adjusting for confounders, the adjusted OR of diabetes comparing extreme quartiles for nickel, copper, arsenic, molybdenum, antimony, zinc and tungsten were 1.653 (95% CIs: 1.035, 2.640), 1.770 (95% CIs: 1.107, 2.831), 1.827 (95% CIs: 1.096, 3.045), 2.003 (95% CIs: 1.222, 3.282), 1.701 (95% CIs: 1.058, 2.734), 4.055 (95% CIs: 2.429, 6.768) and 1.678 (95% CIs: 1.066, 2.640) respectively. Also, the adjusted OR increased with increasing concentration of nickel, copper, arsenic, selenium, molybdenum, antimony, zinc and tungsten per quartiles (all *P*< 0.05). After additional adjustment for multiple hypotheses testing, however, only the dose-response associations of diabetes risk with urinary zinc, molybdenum and tungsten were significant (all FDR-adjusted *P*< 0.05).

**Table 3:** Adjusted OR and 95% CIs of diabetes risk by quartiles of urinary metals.

Variables	Q1 (Lowest)	Q2	Q3	Q4 (Highest)	*P*	*P* *
**Aluminium**	< 21.29	21.29–31.57	31.58–49.22	> 49.22		
N (cases/control)	48/506	60/506	52/506	58/506		
Adjusted OR (95%CIs)	1.000	1.299 (0.850, 1.986)	1.229 (0.794, 1.905)	1.486 (0.967, 2.284)	0.103	0.247
**Titanium**	< 26.08	26.08–45.23	45.24–72.65	> 72.65		
N (cases/control)	45/507	63/505	50/506	50/506		
Adjusted OR (95%CIs)	1.000	1.203 (0.798, 1.812)	0.919 (0.593, 1.426)	1.040 (0.657, 1.645)	0.802	0.878
**Vanadium**	< 0.34	0.34–0.49	0.50–0.74	> 0.74		
N (cases/control)	67/506	52/506	52/506	47/506		
Adjusted OR (95%CIs)	1.000	0.735 (0.487, 1.110)	0.831 (0.549, 1.257)	0.772 (0.500, 1.191)	0.334	0.513
**Chromium**	< 0.94	0.94–1.43	1.44–2.26	> 2.26		
N (cases/control)	62/506	53/506	55/506	48/506		
Adjusted OR (95%CIs)	1.000	0.921 (0.610, 1.389)	0.954 (0.634, 1.435)	0.921 (0.604, 1.406)	0.750	0.862
**Manganese**	< 1.56	1.56–2.44	2.45–3.74	> 3.74		
N (cases/control)	46/506	60/506	53/516	59/496		
Adjusted OR (95%CIs)	1.000	1.238 (0.809, 1.895)	1.152 (0.744, 1.782)	1.318 (0.857, 2.027)	0.285	0.513
**Iron**	< 43.63	43.63–74.68	74.69–140.00	> 140.00		
N (cases/control)	42/506	53/506	71/506	52/506		
Adjusted OR (95%CIs)	1.000	1.186 (0.758, 1.854)	1.459 (0.950, 2.240)	1.190 (0.754, 1.876)	0.318	0.513
**Cobalt**	< 0.16	0.16–0.24	0.25–0.41	> 0.41		
N (cases/control)	49/505	65/507	58/506	46/506		
Adjusted OR (95%CIs)	1.000	1.487 (0.972, 2.274)	1.387 (0.877, 2.194)	1.586 (0.969, 2.597)	0.108	0.247
**Nickel**	< 1.44	1.44–2.24	2.25–3.50	> 3.50		
N (cases/control)	40/506	55/507	59/505	64/506		
Adjusted OR (95%CIs)	1.000	1.406 (0.895, 2.209)	1.528 (0.971, 2.403)	1.653 (1.035, 2.640)	0.040	0.114
**Copper**	< 5.16	5.16–7.31	7.32–10.53	> 10.53		
N (cases/control)	42/506	50/506	49/506	77/506		
Adjusted OR (95%CIs)	1.000	1.072 (0.675, 1.704)	1.124 (0.697, 1.813)	1.770 (1.107, 2.831)	0.012	0.071
**Zinc**	< 164.13	164.13–260.59	260.60–393.36	> 393.36		
N (cases/control)	28/506	23/506	61/505	106/506		
Adjusted OR (95%CIs)	1.000	0.842 (0.468, 1.515)	2.328 (1.410, 3.844)	4.055 (2.429, 6.768)	< 0.001	< 0.001
**Arsenic**	< 16.85	16.85–28.00	28.01–45.98	> 45.98		
N (cases/control)	40/506	53/506	56/506	69/506		
Adjusted OR (95%CIs)	1.000	1.225 (0.774, 1.938)	1.426 (0.884, 2.301)	1.827 (1.096, 3.045)	0.017	0.080
**Selenium**	< 4.53	4.53–7.41	7.42–11.55	> 11.55		
N (cases/control)	48/506	46/506	55/506	69/506		
Adjusted OR (95%CIs)	1.000	0.947 (0.599, 1.497)	1.244 (0.787, 1.968)	1.574 (0.968, 2.559)	0.033	0.108
**Rubidium**	< 1180.93	1180.93–1966.42	1966.43–3060.03	> 3060.03		
N (cases/control)	52/506	64/506	57/506	45/506		
Adjusted OR (95%CIs)	1.000	1.012 (0.660, 1.552)	1.043 (0.659, 1.649)	0.877 (0.507, 1.518)	0.719	0.862
**Strontium**	< 76.11	76.11–123.28	123.29–180.84	> 180.84		
N (cases/control)	60/506	58/506	54/506	46/506		
Adjusted OR (95%CIs)	1.000	1.076 (0.714, 1.622)	1.046 (0.684, 1.599)	0.997 (0.634, 1.566)	0.969	0.969
**Molybdenum**	< 27.21	27.21–45.22	45.23–76.67	> 76.67		
N (cases/control)	38/506	50/506	58/506	72/506		
Adjusted OR (95%CIs)	1.000	1.322 (0.832, 2.101)	1.456 (0.913, 2.322)	2.003 (1.222, 3.282)	0.006	0.049
**Cadmium**	< 0.52	0.52–0.88	0.89–1.43	> 1.43		
N (cases/control)	45/506	63/506	58/506	52/506		
Adjusted OR (95%CIs)	1.000	1.473 (0.947, 2.292)	1.275 (0.796, 2.042)	1.383 (0.817, 2.341)	0.382	0.516
**Tin**	< LOQ	LOQ—0.30	0.31–0.44	> 0.44		
N (cases/control)	84/769	46/414	41/427	47/414		
Adjusted OR (95%CIs)	1.000	1.050 (0.698, 1.579)	1.047 (0.682, 1.610)	1.297 (0.850, 1.980)	0.280	0.513
**Antimony**	< 0.11	0.11–0.16	0.17–0.23	> 0.23		
N (cases/control)	45/505	48/507	52/507	73/505		
Adjusted OR (95%CIs)	1.000	1.110 (0.707, 1.745)	1.175 (0.741, 1.865)	1.701 (1.058, 2.734)	0.028	0.108
**Barium**	< 2.55	2.55–3.80	3.81–5.78	> 5.78		
N (cases/control)	68/506	53/507	48/505	49/506		
Adjusted OR (95%CIs)	1.000	0.825 (0.550, 1.238)	0.808 (0.533, 1.225)	0.911 (0.598, 1.389)	0.609	0.778
**Tungsten**	< 0.07	0.07–0.11	0.12–0.21	> 0.21		
N (cases/control)	44/505	45/507	68/506	61/506		
Adjusted OR (95%CIs)	1.000	1.165 (0.737, 1.840)	1.861 (1.207, 2.869)	1.678 (1.066, 2.640)	0.006	0.049
**Thallium**	< 0.33	0.33–0.56	0.57–0.87	> 0.87		
N (cases/control)	63/506	70/506	47/506	38/506		
Adjusted OR (95%CIs)	1.000	1.118 (0.745, 1.676)	0.856 (0.542, 1.352)	0.841 (0.500, 1.412)	0.325	0.513
**Lead**	< 2.12	2.12–3.18	3.19–4.52	> 4.52		
N (cases/control)	51/506	60/506	48/506	59/506		
Adjusted OR (95%CIs)	1.000	1.049 (0.688, 1.601)	0.890 (0.565, 1.402)	1.290 (0.825, 2.017)	0.381	0.516
**Uranium**	< 0.020	0.020–0.030	0.031–0.046	> 0.046		
N (cases/control)	46/504	63/506	48/510	61/504		
Adjusted OR (95%CIs)	1.000	1.254 (0.818, 1.923)	0.897 (0.567, 1.422)	1.150 (0.734, 1.803)	0.919	0.960

All models were adjusted for age, gender, BMI, smoking status, pack year, alcohol status, family history of diabetes, hypertension, hyperlipidemia and urinary creatinine.

*FDR-adjusted.

### Urinary metals and IFG

Table 4 shows the associations between IFG and concentrations of urinary metals. Participants with urinary aluminum, titanium, vanadium, manganese, strontium, barium and lead above the lowest quartiles had higher IFG concentrations than those in the first quartiles, respectively. As compared to the reference quartile (first quartile), the OR of IFG in the third quartiles of aluminum, vanadium and manganese were 1.638 (95% CIs: 1.114, 2.408), 1.492 (95% CIs: 1.005, 2.214) and 1.480 (95% CIs: 1.012, 2.166) respectively; the OR of IFG in the fourth quartiles of titanium, barium and lead were 1.507 (95% CIs: 1.008, 2.253), 1.549 (95% CIs: 1.048, 2.288) and 1.973 (95% CIs: 1.295, 3.007) respectively; the OR of IFG in the second quartile of strontium were 1.506 (95% CIs: 1.023, 2.218) respectively. Moreover, we observed significant dose-response associations of titanium, zinc, rubidium, barium, tungsten and lead with IFG (all *P*< 0.05). However, only the association between IFG and the quartiles of urinary lead was significant after additionally adjusting for multiple testing (FDR-adjusted *P* = 0.023). We did not observe significant associations of IFG with urinary chromium, iron, cobalt, nickel, copper, arsenic, selenium, molybdenum, cadmium, tin, antimony, thallium and uranium.

**Table 4:** Adjusted OR and 95% CIs of IFG risk by quartiles of urinary metals.

Variables	Q1 (Lowest)	Q2	Q3	Q4 (Highest)	*P*	*P* *
**Aluminium**	< 21.08	21.08–31.19	31.20–49.43	> 49.43		
N (cases/control)	56/441	59/441	82/442	62/441		
Adjusted OR (95%CIs)	1.000	1.128 (0.751, 1.692)	1.638 (1.114, 2.408)	1.245 (0.830, 1.867)	0.107	0.308
**Titanium**	< 25.93	25.93–44.55	45.56–71.08	> 71.08		
N (cases/control)	61/441	53/441	64/442	81/441		
Adjusted OR (95%CIs)	1.000	0.885 (0.587, 1.335)	1.055 (0.707, 1.575)	1.507 (1.008, 2.253)	0.026	0.166
**Vanadium**	< 0.34	0.34–0.49	0.50–0.74	> 0.74		
N (cases/control)	55/441	64/442	77/441	63/441		
Adjusted OR (95%CIs)	1.000	1.147 (0.766, 1.717)	1.492 (1.005, 2.214)	1.207 (0.798, 1.827)	0.215	0.494
**Chromium**	< 0.94	0.94–1.42	1.43–2.23	> 2.23		
N (cases/control)	62/441	62/441	66/442	69/441		
Adjusted OR (95%CIs)	1.000	1.083 (0.733, 1.602)	1.075 (0.729, 1.584)	1.260 (0.857, 1.854)	0.270	0.504
**Manganese**	< 1.54	1.54–2.44	2.45–3.78	> 3.78		
N (cases/control)	56/441	71/441	81/442	51/441		
Adjusted OR (95%CIs)	1.000	1.272 (0.862, 1.876)	1.480 (1.012, 2.166)	0.943 (0.620, 1.433)	0.943	0.943
**Iron**	< 43.68	43.68–75.13	75.14–140.88	> 140.88		
N (cases/control)	68/441	69/441	61/442	61/441		
Adjusted OR (95%CIs)	1.000	0.986 (0.678, 1.435)	0.822 (0.557, 1.212)	0.885 (0.597, 1.312)	0.384	0.519
**Cobalt**	< 0.16	0.16–0.24	0.25–0.41	> 0.41		
N (cases/control)	70/441	59/441	71/442	59/441		
Adjusted OR (95%CIs)	1.000	0.858 (0.576, 1.277)	1.088 (0.727, 1.629)	1.222 (0.791, 1.890)	0.236	0.494
**Nickel**	< 1.44	1.44–2.24	2.25–3.44	> 3.44		
N (cases/control)	62/441	70/442	50/441	77/441		
Adjusted OR (95%CIs)	1.000	1.128 (0.768, 1.658)	0.791 (0.519, 1.206)	1.268 (0.847, 1.899)	0.525	0.636
**Copper**	< 5.14	5.14–7.20	7.21–10.38	> 10.38		
N (cases/control)	55/441	52/441	68/442	84/441		
Adjusted OR (95%CIs)	1.000	0.786 (0.512, 1.205)	1.154 (0.760, 1.752)	1.242 (0.811, 1.900)	0.106	0.308
**Zinc**	< 161.82	161.82–254.79	254.80–385.16	> 385.16		
N (cases/control)	46/441	55/442	69/441	89/441		
Adjusted OR (95%CIs)	1.000	1.098 (0.711, 1.696)	1.396 (0.909, 2.146)	1.538 (0.974, 2.428)	0.036	0.166
**Arsenic**	< 16.82	16.82–28.07	28.08–45.73	> 45.73		
N (cases/control)	63/441	69/442	56/441	71/441		
Adjusted OR (95%CIs)	1.000	0.893 (0.603, 1.323)	0.726 (0.470, 1.120)	0.944 (0.597, 1.493)	0.647	0.709
**Selenium**	< 4.51	4.51–7.37	7.38–11.37	> 11.37		
N (cases/control)	59/441	61/441	58/442	81/441		
Adjusted OR (95%CIs)	1.000	0.944 (0.627, 1.422)	0.930 (0.605, 1.430)	1.380 (0.880, 2.162)	0.161	0.411
**Rubidium**	< 1188.18	1188.18–2010.42	2010.43–3077.77	> 3077.77		
N (cases/control)	68/441	81/441	56/442	54/441		
Adjusted OR (95%CIs)	1.000	0.945 (0.645, 1.384)	0.679 (0.444, 1.038)	0.653 (0.400, 1.066)	0.034	0.166
**Strontium**	< 75.31	75.31–123.95	123.96–180.33	> 180.33		
N (cases/control)	55/441	81/442	55/441	68/441		
Adjusted OR (95%CIs)	1.000	1.506 (1.023, 2.218)	1.111 (0.727, 1.697)	1.400 (0.920, 2.129)	0.341	0.519
**Molybdenum**	< 27.47	27.47–45.08	45.09–74.52	> 74.52		
N (cases/control)	72/441	53/441	52/442	82/441		
Adjusted OR (95%CIs)	1.000	0.697 (0.468, 1.038)	0.681 (0.450, 1.028)	1.111 (0.736, 1.679)	0.646	0.709
**Cadmium**	< 0.53	0.53–0.89	0.90–1.44	> 1.44		
N (cases/control)	67/441	65/441	68/442	59/441		
Adjusted OR (95%CIs)	1.000	0.892 (0.599, 1.326)	0.902 (0.592, 1.374)	0.816 (0.509, 1.308)	0.444	0.567
**Tin**	< LOQ	LOQ—0.30	0.31–0.43	> 0.43		
N (cases/control)	95/674	57/360	50/371	57/360		
Adjusted OR (95%CIs)	1.000	1.119 (0.772, 1.623)	0.995 (0.672, 1.474)	1.258 (0.852, 1.858)	0.364	0.519
**Antimony**	< 0.11	0.11–0.16	0.17–0.23	> 0.23		
N (cases/control)	64/441	68/441	54/442	73/441		
Adjusted OR (95%CIs)	1.000	1.021 (0.693, 1.504)	0.760 (0.499, 1.158)	1.037 (0.674, 1.594)	0.819	0.857
**Barium**	< 2.53	2.53–3.78	3.79–5.74	> 5.74		
N (cases/control)	57/441	65/442	58/441	79/441		
Adjusted OR (95%CIs)	1.000	1.168 (0.785, 1.737)	1.050 (0.698, 1.581)	1.549 (1.048, 2.288)	0.048	0.183
**Tungsten**	< 0.07	0.07–0.11	0.12–0.21	> 0.21		
N (cases/control)	66/441	46/442	71/441	76/441		
Adjusted OR (95%CIs)	1.000	0.772 (0.509, 1.169)	1.165 (0.792, 1.715)	1.382 (0.932, 2.052)	0.036	0.166
**Thallium**	< 0.33	0.33–0.56	0.57–0.88	> 0.88		
N (cases/control)	66/441	55/442	77/441	61/441		
Adjusted OR (95%CIs)	1.000	0.755 (0.498, 1.144)	1.239 (0.828, 1.853)	1.055 (0.667, 1.668)	0.308	0.506
**Lead**	< 2.05	2.05–3.13	3.14–4.44	> 4.44		
N (cases/control)	45/441	61/441	65/442	88/441		
Adjusted OR (95%CIs)	1.000	1.208 (0.790, 1.849)	1.376 (0.894, 2.118)	1.973 (1.295, 3.007)	0.001	0.023
**Uranium**	< 0.020	0.020–0.030	0.031–0.046	> 0.046		
N (cases/control)	58/438	61/446	62/440	78/441		
Adjusted OR (95%CIs)	1.000	1.000 (0.668, 1.495)	0.957 (0.634, 1.443)	1.257 (0.840, 1.882)	0.285	0.504

Abbreviation: IFG, impaired fasting glucose. All models were adjusted for age, gender, BMI, smoking status, pack year, alcohol status, hypertension, hyperlipidemia, family history of diabetes, anti-diabetes drug, insulin use and urinary creatinine.

*FDR-adjusted.

### Sensitivity analysis

Severe diabetes might have been accompanied by renal impairment, which may cause abnormal urine excretion of trace elements and heavy metals. We thus conducted sensitivity analyses according to the diabetic status. The results are presented in S2 Table. We found that diabetes was dose-dependently associated with urinary cooper, zinc, arsenic, selenium, molybdenum and tungsten after we excluded the participants who were using insulin treatment (*P*< 0.05). After additionally adjusting for multiple testing, diabetes was related to urinary zinc only (FDR-adjusted *P*< 0.05). Urinary nickel, copper and zinc were dose-dependently associated with diabetes risk when we restricted the analysis to participants without previously diagnosed diabetes and a history of anti-diabetic drug use. However, only the dose-response association between diabetes risk and urinary zinc was significant after additionally adjusting for multiple comparisons (FDR-adjusted *P*< 0.05).

## Discussion

Exposure to heavy metal, mainly via dietary intake, drinking water, and inhalation of air particles, is a major public health problem, especially in China. In the current study, we found that multiple metals in urine are associated with FPG, IFG or diabetes risk among a general Chinese population.

### Toxic heavy metals

We found a significant association between urinary arsenic and diabetes risk among the general Chinese population, which was in accordance with previous findings of increased diabetes risk in general U.S. population with exposure to arsenic [5,8]. Experimental evidence suggested that arsenic could impair insulin synthesis and secretion in pancreatic β-cells and decrease glucose uptake in insulin sensitive cells [44,45]. We also found that arsenic remained associated with increased risk of diabetes after the subjects using insulin were excluded from the analyses, suggesting that association between urinary arsenic and diabetes risk in the population should not be regarded as of doubtful significance. However, no association was observed between urinary arsenic and newly recognized diabetes (diagnosed by FPG levels, S2 Table), indicating that diabetic status may modify the association between urinary arsenic and diabetes risk. Nonetheless, the exact reasons for the difference are unclear and deserve further investigation.

Findings from in vitro and animal studies indicated that cadmium could cause diabetes mellitus through the induction of oxidative stress and disruption of pancreatic β-cell function [46]. However, our results suggested that urinary cadmium was not related to diabetes, which was in line with most previous studies [12,13] but not all [11]. The discrepancy between current and previous epidemiological investigation which found a significant association between urinary cadmium and diabetes risk may be due to a lower prevalence of diabetes in our population compared to the US study population.

We found that antimony was associated with increased diabetes risk but the association attenuated to null after participants with insulin and other anti-hyperglycemia drug uses as well as subjects with a history of self-reported diabetes were excluded from the analysis. This suggested that the estimates of association between urinary antinomy and diabetes risk may depend on the diabetic status. Prospective cohort study design in the future is warranted.

We also found that urinary lead was associated with elevated FPG and IFG. Few epidemiological studies investigated the potential relationships of lead exposure with FPG and diabetes risk. Previous studies have shown that blood lead levels among diabetics were higher than that among controls [47,48] but a recently epidemiological study failed to find a significant association between blood lead and diabetes risk among general Korean population [13]. Urinary lead has been recommended as a good biomarker of lead exposure among population groups [49]. Experimental evidence has suggested that lead may contribute to an abnormal glucose metabolism by inhibiting the reabsorption of glucose through damage in the proximal renal tubule [50].

### Trace elements

We found that iron was associated with decreased FPG among subjects in the fourth quartile compared with those in the first quartile of iron, which is consistent with previous studies [51,52]. However, iron was not related to decreased risks of diabetes and IFG, which suggested that current intake levels of iron may not benefit the disorders of glucose metabolism.

In the present study, higher urinary nickel level was significantly associated with higher risk of FPG and diabetes. No prior epidemiological study has been conducted to investigate the association of nickel exposure and diabetes risk. However, it has been reported that body burden of nickel might be altered in diabetics, but the results were inconsistent. For instance, Yarat et al. [53] found a lower serum nickel concentration in diabetes patients, whereas Kazi et al. [19] showed no difference in blood levels of nickel between patients with diabetes and healthy controls. Moreover, Aguilar et al. [54] reported a higher concentration of plasma nickel in diabetics.

The physiological role of nickel, an essential element for mammals, has not been completely understood. A previous animal study revealed that nickel could increase plasma glucose levels by disruption of glucose homeostasis and induction of insulin resistance [55]. However, the sensitivity analyses in the present study indicated that there was no association between nickel in the fourth quartile and diabetes risk. Therefore, further studies are needed to clarify this.

Copper, the third most abundant essential transition metal in human body, was associated with increased FPG levels and diabetes risk in the current analyses. As a cofactor of several enzymes, Copper is involved in a number of physiological pathways as structure components and its overload has been associated with neurodegenerative diseases such as Alzheimer disease. Copper could play an important role in the pathogenesis of diabetes including the facilitation of hydrogen peroxide generation from amylin, and the induction of degeneration and death of pancreatic islet cells [56].

Our study also indicated that urinary zinc was strongly correlated with increased FPG and risk of IFG and diabetes, which is consistent with a previous study [19]. Zinc supplementation can be effective for preventing or ameliorating diabetes. Zinc transporter (ZnT-8) is a crucial protein for the regulation of insulin secretion in pancreatic β-cells [57]. Several studies have reported that diabetics had lower serum/plasma zinc levels [54,58]. Especially, a recent case-control study suggested that plasma zinc was associated with lower risk of type 2 diabetes [59]. It has been postulated that hyperglycemia interferes with the active transport of zinc back into the renal tubular cells, and thus results in loss of a large amount of zinc from the body via the urine of individuals with diabetes [60]. Our findings support the possibility that increased urinary excretion of zinc suggests a deficiency in blood zinc and further dysregulation of insulin secretion [61].

Selenium is incorporated into selenoproteins that have a wide range of pleiotropic effects. Conflicting evidence linking selenium to glucose metabolism has been reported. For instance, high selenium status was associated with reduced diabetes prevalence in several prospective cohort and case-control studies [22,62]. However, high serum and plasma selenium concentrations were associated with an increased prevalence of diabetes and FPG in the large U.S. National Health and Nutrition Examination Surveys [21] and the French SUVIMAX trial population [63]. Consistently, our results showed that elevated urinary selenium levels were significantly correlated with increased FPG and diabetes risk. The increased risk of diabetes with selenium exposure might be explained by selenium initiating an insulin signaling cascade accompanied by a burst of hydrogen peroxide [64].

There is accumulating evidence that molybdenum plays an important role in insulin action as it has been suggested to be able to bind the insulin receptor and to be involved in the activation of glucose metabolism enzymes [18]. Therefore, molybdenum complexes have been proposed as possible adjunct in the treatment of diabetes mellitus [65]. On the contrary, we found that elevated molybdenum was correlated with increased diabetes risk in the general Chinese population whereas no significant correlation was seen among the population after excluding the participants with insulin use as well as previously diagnosed diabetes and a history of anti-diabetic drug use. Thus, we speculated that the significant findings in the present study may be the result of chance alone.

### Metals with unknown biological function or toxicity

Little is known regarding the biological roles, toxicity and carcinogenicity of aluminum, titanium, rubidium, strontium, barium and tungsten in humans. In the present study, we found that these metals were associated with one or more diabetes-related indices. Because there was no a priori specific hypothesis about how these metals associated with diabetes risk, coupled with the fact that no plausible mechanism for glucose effects has been postulated, our results warrant further investigation.

### Limitations

Firstly, we do not know whether diabetes results from elevated metals levels or vice versa because the cross-sectional design hinders us to draw inferences regarding causality. Secondly, the dataset prevents us from differentiating type 1 from type 2 diabetes, and the association of metals with diabetes might differ by diabetes type. However, most subjects are likely to have type 2 diabetes because there were only 2 subjects aged less than 40 years in this population. Thirdly, there may be independent measurement errors in this study since multiple metals were examined in the same urine sample by the same assay, which may result in potentially misleading findings [66]. Fourthly, some findings obtained in the present study may be by chance because of the multiple tests. Finally, as our results were obtained only by the urinary output of these metals, we cannot exclude the possibility of a false positive. Therefore, the associations found in this study need to be further investigated in future studies.

## Conclusions

The present study found that urinary vanadium, chromium, manganese, cobalt, cadmium, tin, barium, thallium and uranium were not associated with FPG concentrations, IFG, or diabetes risk; whereas urinary titanium, nickel, copper, zinc, arsenic, selenium, rubidium, molybdenum, antimony, barium, tungsten and lead were dose-dependently related to one or more diabetes-related outcomes. Because no prior study has examined the association of multiple metals with FPG or diabetes, in addition to the potential for misleading findings due to multiple comparisons as well as independent measurement errors, the present findings need replication in future studies with large enough sample sizes.

## Supporting Information

S1 Table The distributions of urinary metals in the community-dwelling population (n = 2242). (DOCX)

S2 Table Sensitivity analysis for the association between urinary metals (Quartiles) and diabetes risk. (DOCX)
