# Supplementary material for: Association of Urinary Metal Profiles with Altered Glucose Levels and Diabetes Risk: A Population-Based Study in China
Source: PLoS One. 2015 Apr 13;10(4):e0123742. doi: 10.1371/journal.pone.0123742 (PMC4395404; doi:10.1371/journal.pone.0123742)
Supplement: S1 Table — (DOCX) [file pone.0123742.s001.docx]

| Table S1. The distributions of urinary metals in the community-dwelling population (n=2242) | | | | | | | | | | | | |
| --- | --- | --- | --- | --- | --- | --- | --- | --- | --- | --- | --- | --- |
| Urinary metals | Unstandardized for urinary creatinine | | | | |  | Urinary creatinine-standardized | | | | | N (%) < LOQ |
|  | Percentile 5th | Percentile 25th | Percentile 50th | Percentile 75th | Percentile 95th |  | Percentile 5th | Percentile 25th | Percentile 50th | Percentile 75th | Percentile 95th |  |
| Aluminium | 11.979 | 21.364 | 31.619 | 49.490 | 117.213 |  | 0.970 | 1.724 | 2.753 | 4.678 | 12.756 | 0 (0.0) |
| Titanium | 11.505 | 26.077 | 44.857 | 72.036 | 135.478 |  | 1.179 | 2.355 | 3.914 | 6.265 | 10.952 | 0 (0.0) |
| Vanadium | 0.189 | 0.338 | 0.488 | 0.740 | 1.356 |  | 0.016 | 0.028 | 0.043 | 0.068 | 0.137 | 0 (0.0) |
| Chromium | 0.498 | 0.935 | 1.421 | 2.221 | 4.364 |  | 0.039 | 0.075 | 0.124 | 0.218 | 0.489 | 2 (0.1) |
| Manganese | 0.788 | 1.567 | 2.448 | 3.754 | 7.494 |  | 0.063 | 0.127 | 0.210 | 0.358 | 0.862 | 0 (0.0) |
| Iron | 21.810 | 44.219 | 75.573 | 139.569 | 374.008 |  | 1.853 | 3.754 | 6.799 | 12.886 | 36.350 | 0 (0.0) |
| Cobalt | 0.079 | 0.156 | 0.240 | 0.404 | 1.167 |  | 0.008 | 0.014 | 0.021 | 0.036 | 0.102 | 2 (0.1) |
| Nickel | 0.758 | 1.479 | 2.255 | 3.524 | 7.541 |  | 0.068 | 0.127 | 0.198 | 0.323 | 0.656 | 7 (0.3) |
| Copper | 2.980 | 5.192 | 7.400 | 10.707 | 19.790 |  | 0.294 | 0.463 | 0.640 | 0.922 | 1.846 | 0 (0.0) |
| Zinc | 84.568 | 168.042 | 270.494 | 412.424 | 816.223 |  | 8.669 | 16.235 | 23.531 | 35.181 | 64.190 | 0 (0.0) |
| Arsenic | 7.106 | 17.166 | 28.434 | 46.465 | 83.363 |  | 0.892 | 1.702 | 2.474 | 3.525 | 6.472 | 0 (0.0) |
| Selenium | 2.130 | 4.553 | 7.489 | 11.761 | 22.202 |  | 0.258 | 0.447 | 0.656 | 0.948 | 1.543 | 0 (0.0) |
| Rubidium | 512.519 | 1187.580 | 1956.851 | 3035.462 | 4975.528 |  | 62.472 | 115.326 | 170.181 | 234.905 | 362.127 | 0 (0.0) |
| Strontium | 35.847 | 75.524 | 122.633 | 178.428 | 310.820 |  | 3.432 | 6.819 | 10.791 | 15.760 | 27.950 | 0 (0.0) |
| Molybdenum | 11.398 | 27.794 | 45.956 | 77.966 | 152.198 |  | 1.331 | 2.704 | 4.114 | 6.212 | 11.787 | 0 (0.0) |
| Cadmium | 0.247 | 0.529 | 0.885 | 1.420 | 2.848 |  | 0.028 | 0.051 | 0.078 | 0.116 | 0.218 | 0 (0.0) |
| Tin | < LOQ | < LOQ | 0.269 | 0.393 | 0.781 |  | < LOQ | < LOQ | 0.022 | 0.034 | 0.074 | 853 (38.0) |
| Antimony | 0.062 | 0.112 | 0.162 | 0.231 | 0.403 |  | 0.006 | 0.010 | 0.014 | 0.020 | 0.036 | 0 (0.0) |
| Barium | 1.455 | 2.522 | 3.775 | 5.767 | 10.794 |  | 0.108 | 0.208 | 0.335 | 0.551 | 1.210 | 0 (0.0) |
| Tungsten | 0.023 | 0.067 | 0.116 | 0.212 | 0.781 |  | 0.002 | 0.006 | 0.010 | 0.019 | 0.062 | 65 (2.9) |
| Thallium | 0.143 | 0.324 | 0.552 | 0.859 | 1.488 |  | 0.017 | 0.032 | 0.047 | 0.069 | 0.119 | 0 (0.0) |
| Lead | 0.446 | 2.129 | 3.175 | 4.534 | 9.030 |  | 0.081 | 0.183 | 0.272 | 0.414 | 0.790 | 125 (5.6) |
| Uranium | 0.010 | 0.020 | 0.030 | 0.047 | 0.085 |  | 0.001 | 0.002 | 0.003 | 0.004 | 0.009 | 15 (0.7) |
