# Supplementary material for: Association of Urinary Metal Profiles with Altered Glucose Levels and Diabetes Risk: A Population-Based Study in China
Source: PLoS One. 2015 Apr 13;10(4):e0123742. doi: 10.1371/journal.pone.0123742 (PMC4395404; doi:10.1371/journal.pone.0123742)
Supplement: S2 Table — (DOCX) [file pone.0123742.s002.docx]

| Table S2. Sensitivity analysis for the association between urinary metals (Quartiles) and diabetes risk. | | | | | | | | | | | | | |
| --- | --- | --- | --- | --- | --- | --- | --- | --- | --- | --- | --- | --- | --- |
| Variables | Diabetes without insulin use vs. (IFG+NGT) | | | | | |  | Undiagnosed diabetes vs. (IFG+NGT) | | | | | |
|  | Q1 (Lowest) | Q2 | Q3 | Q4 (Highest) | *P* | *P*^*^ |  | Q1 (Lowest) | Q2 | Q3 | Q4 (Highest) | *P* | *P*^*^ |
| **Aluminium** | 1.000 | 1.375 (0.891, 2.121) | 1.173 (0.744, 1.851) | 1.482 (0.952, 2.307) | 0.156 | 0.397 |  | 1.000 | 2.421 (0.998, 5.873) | 2.769 (1.171, 6.549) | 2.348 (0.959, 5.748) | 0.075 | 0.348 |
| **Titanium** | 1.000 | 1.158 (0.759, 1.766) | 0.979 (0.628, 1.526) | 0.988 (0.614, 1.587) | 0.773 | 0.847 |  | 1.000 | 1.221 (0.578, 2.576) | 0.797 (0.351, 1.814) | 1.104 (0.499, 2.443) | 0.908 | 0.908 |
| **Vanadium** | 1.000 | 0.768 (0.506, 1.166) | 0.807 (0.527, 1.236) | 0.726 (0.463, 1.141) | 0.203 | 0.468 |  | 1.000 | 0.991 (0.496, 1.980) | 0.552 (0.241, 1.263) | 0.733 (0.341, 1.573) | 0.228 | 0.425 |
| **Chromium** | 1.000 | 0.860 (0.565, 1.307) | 0.910 (0.601, 1.377) | 0.820 (0.529, 1.271) | 0.437 | 0.669 |  | 1.000 | 0.802 (0.390, 1.653) | 0.691 (0.326, 1.464) | 0.917 (0.443, 1.897) | 0.708 | 0.779 |
| **Manganese** | 1.000 | 1.227 (0.792, 1.899) | 1.172 (0.750, 1.831) | 1.252 (0.802, 1.953) | 0.395 | 0.669 |  | 1.000 | 1.726 (0.764, 3.899) | 1.586 (0.702, 3.584) | 1.850 (0.814, 4.204) | 0.201 | 0.425 |
| **Iron** | 1.000 | 1.206 (0.767, 1.896) | 1.411 (0.910, 2.186) | 1.067 (0.665, 1.712) | 0.634 | 0.782 |  | 1.000 | 0.936 (0.421, 2.082) | 1.147 (0.537, 2.451) | 1.187 (0.549, 2.568) | 0.557 | 0.712 |
| **Cobalt** | 1.000 | 1.355 (0.881, 2.084) | 1.244 (0.780, 1.982) | 1.379 (0.832, 2.287) | 0.293 | 0.561 |  | 1.000 | 1.287 (0.572, 2.898) | 1.624 (0.719, 3.668) | 1.830 (0.749, 4.470) | 0.151 | 0.425 |
| **Nickel** | 1.000 | 1.327 (0.842, 2.094) | 1.410 (0.891, 2.231) | 1.429 (0.886, 2.304) | 0.155 | 0.397 |  | 1.000 | 1.339 (0.551, 3.255) | 1.724 (0.727, 4.085) | 2.504 (1.051, 5.966) | 0.026 | 0.196 |
| **Copper** | 1.000 | 0.944 (0.587, 1.518) | 0.981 (0.601, 1.600) | 1.672 (1.042, 2.684) | 0.020 | 0.094 |  | 1.000 | 2.403 (0.852, 6.781) | 1.653 (0.542, 5.043) | 3.643 (1.280, 10.369) | 0.023 | 0.196 |
| **Zinc** | 1.000 | 0.762 (0.418, 1.389) | 2.123 (1.278, 3.526) | 3.674 (2.188, 6.168) | < 0.001 | < 0.001 |  | 1.000 | 0.405 (0.099, 1.662) | 2.520 (0.952, 6.673) | 3.380 (1.247, 9.163) | < 0.001 | 0.009 |
| **Arsenic** | 1.000 | 1.247 (0.777, 2.002) | 1.379 (0.840, 2.265) | 1.886 (1.116, 3.187) | 0.017 | 0.094 |  | 1.000 | 1.073 (0.699, 4.151) | 1.383 (0.533, 3.593) | 2.504 (0.950, 6.597) | 0.099 | 0.348 |
| **Selenium** | 1.000 | 0.866 (0.540, 1.387) | 1.157 (0.723, 1.850) | 1.536 (0.937, 2.516) | 0.042 | 0.161 |  | 1.000 | 0.604 (0.254, 1.437) | 1.005 (0.437, 2.310) | 1.440 (0.607, 3.412) | 0.174 | 0.425 |
| **Rubidium** | 1.000 | 0.925 (0.597, 1.434) | 0.970 (0.608, 1.549) | 0.835 (0.477, 1.461) | 0.617 | 0.782 |  | 1.000 | 0.979 (0.465, 2.064) | 0.721 (0.308, 1.685) | 0.620 (0.235, 1.632) | 0.247 | 0.425 |
| **Strontium** | 1.000 | 1.097 (0.717, 1.677) | 1.122 (0.725, 1.735) | 1.039 (0.651, 1.656) | 0.838 | 0.877 |  | 1.000 | 1.506 (0.621, 3.652) | 2.407 (1.039, 5.577) | 1.943 (0.799, 4.722) | 0.090 | 0.348 |
| **Molybdenum** | 1.000 | 1.323 (0.824, 2.126) | 1.379 (0.852, 2.230) | 2.019 (1.218, 3.348) | 0.008 | 0.076 |  | 1.000 | 1.436 (0.648, 3.183) | 1.165 (0.504, 2.694) | 1.306 (0.550, 3.104) | 0.711 | 0.779 |
| **Cadmium** | 1.000 | 1.368 (0.871, 2.147) | 1.123 (0.692, 1.823) | 1.369 (0.803, 2.333) | 0.432 | 0.669 |  | 1.000 | 1.893 (0.810, 4.424) | 1.467 (0.581, 3.707) | 1.771 (0.661, 4.741) | 0.446 | 0.604 |
| **Tin** | 1.000 | 1.019 (0.724, 1.643) | 0.979 (0.629, 1.526) | 1.139 (0.732, 1.773) | 0.680 | 0.782 |  | 1.000 | 1.489 (0.733, 3.023) | 1.362 (0.650, 2.857) | 1.175 (0.522, 2.645) | 0.638 | 0.772 |
| **Antimony** | 1.000 | 1.045 (0.657, 1.661) | 1.162 (0.727, 1.857) | 1.556 (0.957, 2.532) | 0.065 | 0.212 |  | 1.000 | 1.155 (0.497, 2.682) | 1.415 (0.610, 3.281) | 1.945 (0.825, 4.583) | 0.106 | 0.348 |
| **Barium** | 1.000 | 0.803 (0.529, 1.218) | 0.814 (0.532, 1.245) | 0.857 (0.554, 1.325) | 0.479 | 0.689 |  | 1.000 | 1.219 (0.549, 2.706) | 1.199 (0.540, 2.664) | 1.504 (0.687, 3.290) | 0.335 | 0.482 |
| **Tungsten** | 1.000 | 1.138 (0.712, 1.817) | 1.783 (1.144, 2.779) | 1.640 (1.033, 2.606) | 0.010 | 0.076 |  | 1.000 | 1.522 (0.676, 3.431) | 1.903 (0.865, 4.185) | 1.561 (0.666, 3.658) | 0.259 | 0.425 |
| **Thallium** | 1.000 | 1.143 (0.756, 1.729) | 0.785 (0.488, 1.263) | 0.837 (0.492, 1.424) | 0.254 | 0.531 |  | 1.000 | 1.212 (0.569, 2.585) | 1.101 (0.484, 2.502) | 0.601 (0.222, 1.626) | 0.306 | 0.470 |
| **Lead** | 1.000 | 0.967 (0.628, 1.490) | 0.886 (0.561, 1.400) | 1.137 (0.718, 1.800) | 0.674 | 0.782 |  | 1.000 | 2.490 (0.968, 6.404) | 1.991 (0.743, 5.336) | 2.373 (0.882, 6.387) | 0.225 | 0.425 |
| **Uranium** | 1.000 | 1.264 (0.815, 1.960) | 0.862 (0.535, 1.389) | 1.156 (0.730, 1.832) | 0.941 | 0.941 |  | 1.000 | 1.216 (0.568, 2.602) | 0.872 (0.387, 1.967) | 1.005 (0.447, 2.261) | 0.777 | 0.812 |
| Abbreviation: NGT, normal glucose tolerance; IFG, impaired fasting glucose. All models were adjusted for age, gender, BMI, smoking status, pack year, alcohol status, family history of diabetes, hypertension, hyperlipidemia and urinary creatinine. | | | | | | | | | | | | | |
